# Monocyte Gene and Molecular Expression Profiles Suggest Distinct Effector and Regulatory Functions in Beninese HIV Highly Exposed Seronegative Female Commercial Sex Workers

**DOI:** 10.3390/v14020361

**Published:** 2022-02-10

**Authors:** Laurence Blondin-Ladrie, Lyvia Fourcade, Alessandro Modica, Matheus Aranguren, Nicolas de Montigny, Annie-Claude Labbé, Michel Alary, Fernand Guédou, Johanne Poudrier, Michel Roger

**Affiliations:** 1 Centre de Recherche du Centre Hospitalier de l’Université de Montréal (CRCHUM), Montreal, QC H2X 0A9, Canada; laurence.blondin-ladrie@umontreal.ca (L.B.-L.); lyvia.fourcade@gmail.com (L.F.); alexacidom@gmail.com (A.M.); mat.aranguren@gmail.com (M.A.); 2 Département de Microbiologie, Infectiologie et Immunologie de l‘Université de Montréal, Montreal, QC H3T 1J4, Canada; ac.labbe@umontreal.ca; 3 Département de Sciences de l’informatique, Université du Québec à Montréal, Montreal, QC H2X 3Y7, Canada; de_montigny.nicolas@courrier.uqam.ca; 4 Service de Maladies Infectieuses, Hôpital Maisonneuve-Rosemont, Montreal, QC H1T 2M4, Canada; 5 Centre de Recherche du CHU de Québec-Université Laval, Quebec, QC G1E 6W2, Canada; michel.alary@crchudequebec.ulaval.ca; 6 Département de Médecine Sociale et Préventive, Université Laval, Quebec, QC G1V 0A6, Canada; 7 Institut National de Santé Publique du Québec, Quebec, QC H2P 1E2, Canada; 8 Dispensaire IST, Cotonou, Benin; guedaf@yahoo.fr

**Keywords:** HIV, resistance, highly exposed seronegative (HESN), commercial sex workers, monocytes, effector functions, regulatory functions

## Abstract

We have previously reported that the female genital tract (FGT) of Beninese HIV highly-exposed seronegative (HESN) commercial sex workers (CSWs), presented elevated frequencies of a myeloid HLA-DR+CD14+CD11c+ population presenting “tolerogenic” monocyte derived dendritic cells (MoDC) features. In order to assess whether a differential profile of monocytes may be involved in the generation of these genital MoDCs, we have herein characterized the blood monocyte compartment of Beninese HESNs (HIV-uninfected ≥ 10 years CSWs) and relevant controls (HIV-uninfected 2.5–5 years CSWs herein termed “early HESNs”), HIV-infected CSWs, and low-risk HIV-uninfected women from the general population. Transcriptomic analyses by RNA-Seq of total sorted blood monocytes demonstrate that in comparison to the control groups, HESNs present increased expression levels of FCGR2C, FCAR, ITGAX, ITGAM, CR2, CD68, and CD163 genes, associated with effector functions. Moreover, we found increased expression levels of genes associated with protection/control against SHIV/HIV such as CCL3, CCL4, CCL5, BHLHE40, and TNFSF13, as well as with immune regulation such as IL-10, Ahr, CD83, and the orphan nuclear receptor (NR)4A1, NR4A2, and NR4A3. Through multicolor flow cytometry analyses, we noticed that the frequencies of intermediate and non-classical monocyte populations tended to be elevated in the blood of HESNs, and exhibited increased expression levels of effector CD16, CD11c, CD11b, as well as regulatory HLA-G, IL-10, and IFN-α markers when compared to HIV-uninfected women and/or HIV-infected CSWs. This profile is compatible with that previously reported in the FGT of HESNs, and likely confers an enormous advantage in their resistance to HIV infection.

## 1. Introduction

Most HIV infections are acquired through heterosexual intercourse, and in sub-Saharan Africa, 59% of new infections affect women [1]. The authors and others have established cohorts of heavily HIV-exposed African female commercial sex workers (CSWs), in which some women remain HIV-uninfected after more than 7 years of active sex work [2]. The study of these HIV-1 highly-exposed seronegative (HESN) women, who constitute a model of natural immunity to HIV, provides an exceptional opportunity to determine important clues for the development of preventive strategies, which at the moment, remain the best solutions to eradicate the pandemic. We have previously reported that Beninese HESN CSWs present a low-inflammatory profile in their female genital tract (FGT) [3,4]. We found this low-inflammatory profile to be concomitant with important antiviral and regulatory features. Notably, Beninese HESNs presented elevated frequencies of an endocervical myeloid HLA-DR+CD14+CD11c+ population, expressing high levels of anti-viral (TLR7, IFN-α) and immunoregulatory (IL-10, HLA-G, ILT4) molecules [5]. Concomitantly, we found elevated frequencies of endocervical T regulatory (Treg) and Tr1 (CD49b+LAG3+) cells, expressing high levels of PD-1 and IL-10, reflective of their active state [6,7]. These are in agreement with the elevated frequencies of Tregs, which was previously reported in the blood of Kenyan HESNs [8]. The HLA-DR+CD14+CD11c+ population described in the FGT of Beninese HESNs, is reminiscent of tolerogenic monocyte derived dendritic cells (MoDCs) or “DC-10” [9], that produce high amounts of IL-10, express high levels of HLA-G and ILT-4, and can induce Tr1 via an IL-10–dependent ILT4/HLA-G pathway [10]. In addition, both IL-10 and IFN-α promote Tr1 differentiation [11]. The increase in frequencies of tolerogenic or “DC-10-like” MoDCs endowed with tolerogenic as well as IL-10- and IFN-α-producing capacities is likely to contribute to the overall protection from HIV in HESNs, by orchestrating potent anti-viral and regulatory activities at a major portal of entry for HIV.

Genital tolerogenic MoDCs are possibly derived from blood monocytes, known to demonstrate developmental plasticity as they differentiate into macrophages, MoDC, or osteoclasts depending on the inflammatory milieu (reviewed in [12]). In humans, monocytes are categorized in 3 main populations based on their expression levels of CD14 and CD16, each representing a stage of differentiation [12]. They are the predominant CD14+CD16- classical, and to a lesser extent CD14+CD16+ intermediate and CD14low/dimCD16++ non-classical populations. Further distinction refers to differential expression levels of chemokine receptors CCR2 and fractalkine receptor CX3CR1. CCR2 is highly expressed by the classical majority, while CX3CR1 is lower at this stage and rises while CCR2 goes down on the non-classical minority. Classical monocytes mainly recirculate or migrate into tissues via CCR2 to address an assault and/or replenish monocyte-derived macrophages and/or MoDC populations, whereas CX3CR1 allows for non-classical monocytes to patrol for endothelial integrity. Studies on mouse Ly6Clow monocytes, which are considered the murine analogues of human non-classical monocytes, whereas Ly6Chi correspond to classical monocytes, demonstrated that the former are enriched within capillaries and scavenge microparticles and necrotic debris from their luminal side in a steady state [13].

Orphan nuclear receptor (NR)4A1 (Nur77), NR4A2 (Nurr1), and NR4A3 (NOR1) are transcriptional regulators of differentiation, proliferation, and apoptosis genes. The transition from classical to intermediate and non-classical monocytes depend on the NR4A1 expression [14], necessary to their generation and survival, as they are reduced by 90% in NR4A1-deficient mice [15]. Furthermore, recent studies of NR4A3-deficient mice show that NR4A3 is required to skew monocyte differentiation toward MoDCs and allows the acquisition of migratory characteristics required for MoDC function [16].

In order to start addressing whether a differential profile of monocytes may be involved in the generation of tolerogenic MoDCs, we have herein characterized the blood monocyte compartment of Beninese HESNs (HIV-uninfected ≥ 10 years CSWs) and compared to that of relevant controls (HIV-uninfected 2.5–5 years CSWs “early HESN” i.e.,: beyond the immune activation generated by ≤1 year sex work [17] and possibly evolving towards a HESN status, HIV-infected CSWs, and low-risk HIV-uninfected women from the general population). The fact that NR4As are pivotal to monocyte differentiation, prompted us to explore expression levels of these factors along with those associated with the anti-viral and regulatory profile that we previously described for genital tolerogenic MoDCs. Based on transcriptomic and multicolor flow cytometry analyses, we report that blood monocytes from HESNs present elevated expression levels of NR4As, as well as anti-viral and regulatory markers when compared to other groups. Moreover, we found increased expression levels of markers associated with effector functions. Altogether, our observations suggest that the differential profile of blood monocytes from HESNs reflects enhanced effector, antiviral, and regulatory functions. This profile is compatible with that of tolerogenic MoDC, previously reported in the FGT of HESNs, and likely confers an enormous advantage to HESNs in their resistance to HIV infection.

## 2. Materials and Methods

**Study populations.** Non-CSW control women at low risk for exposure were enrolled from a general health clinic in Cotonou, Benin. Female CSWs were recruited through a dedicated sex worker clinic in Cotonou. Women were invited to participate in the study as they attended clinics. Women were excluded from the study if they were less than 18 years old, menstruating, or pregnant. At enrolment, participants were asked to answer a questionnaire about demographic information, sexual behavior, duration of sex work, number of sex partners, condom use, vaginal douching practices, and reproductive history. Each participant underwent a genital examination by a physician. Vaginal specimens were obtained for diagnosis of candidiasis, trichomoniasis, and bacterial vaginosis by microscopic examination and HSV infection by PCR. Endocervical swabs were obtained to test for Neisseria gonorrhoea and Chlamydia trachomatis infection using the BD ProbeTec ET system (Strand Displacement Assay, Becton Dickinson, Heidelberg, Germany). Peripheral blood was taken for HIV, syphilis, HSV, and progesterone testing by immunoassays. HIV-1 positivity was defined by the presence of HIV-1-specific IgG tested with Vironostika HIV Uni-Form II Ag/Ab (Organon Teknika, Boxtel, the Netherlands). Non-reactive samples were considered HIV seronegative, whereas reactive samples were tested with Genie II HIV-1/HIV-2 (Bio-Rad, Hercules, CA, USA). Genie II dually-reactive samples (to HIV-1 and HIV-2) and discordant samples (Vironostika reactive/Genie II non-reactive) were further tested by INNO-LIA HIV I/II Score (Innogenetics NV, Technologiepark 6, Gent, Belgium). HSV infection and shedding was determined by testing for HSV-specific antibodies in the serum and for the presence of HSV in the CVLs of the women by PCR assay. Of note, the participants of this study were primarily tested for sexually transmitted diseases and not for HBV, HCV, or HTLV, which are commonly associated with high-risk groups. However, we feel any influence might be comparable amongst our study groups. In the present study, samples were selected from 9 HESNs (HIV-1-uninfected ≥ 10 years CSWs), 11 HIV-1-uninfected 2.5–5 years CSWs “early HESNs”, 10 HIV-1-infected CSWs, and 7 HIV-1-uninfected non-CSW control women from the general population for characterization by flow cytometry of blood monocytes. For the transcriptomic analyses of sorted total blood monocytes, samples from 4 HESNs and 3 from other groups were selected. The four study groups were all in the follicular phase of their menstrual cycle as determined by progesterone levels, not taking oral contraception, and had no co-infection, bacterial vaginosis, trichomoniasis, or candidiasis.

**Ethics statement.** Written informed consent was obtained from all subjects who participated in the study. The methods reported in this paper were performed in accordance with the relevant guidelines and regulations and all experimental protocols were approved by the Comité National Provisoire d’Éthique de la Recherche en Santé in Cotonou and the Centre Hospitalier de l’Université de Montréal (CHUM) Research Ethics Committees.

**Sample collection and preparation.** Peripheral blood mononuclear cells (PBMCs) were isolated from whole blood by centrifugation on Ficoll gradients, washed and suspended in freezing medium (90% heat inactivated fetal bovine serum (hi-FBS), 10% dimethyl sulfoxyde (DMSO)), and kept in liquid nitrogen until use. Plasma and serum were kept frozen at −80 °C until use.

**Multicolor Flow Cytometry Analyzes.** PBMCs samples were thawed, washed in IMDM followed by 1× PBS, and processed for flow cytometry. Briefly, each sample of PBMCs was separated in two for intracellular and intranuclear staining. Live/dead exclusion was performed using Aqua-LIVE/DEAD Fixable Stain (Invitrogen Life technologies, Eugene, OR, USA). Non-specific binding sites were blocked using fluorescence-activated cell sorting (FACS) buffer (1× PBS, 2% hi-FBS, and 0.1% sodium azide) supplemented with 20% hi-FBS, and 10 μg of mouse and/or rat IgG (Sigma-Aldrich, St-Louis, MO, USA) and 8 μg Human BD FCBlock (BD Biosciences) per million cells. The following conjugated mouse and/or rat anti-human monoclonal antibodies were used for the detection of surface markers: anti-HLA-G-PerCP-eFluor710 (eBiosciences), anti-HLA-DR-BB515, anti-CD16-BUV737, anti-CD14-BV786, anti-CD123-BV711, anti-CD83-BV650, anti-CD11b-BUV395, anti-CCR2-BV421, rat-anti-CX3CR1- BV421 (BD Biosciences), and anti-CD11c-Pe/Cy5.5 (Invitrogen by Thermo Fisher Scientific), anti-ILT4-AF594 (R&D systems). For the detection of intracellular markers, the following conjugated mouse and/or rat anti-human monoclonal antibodies were used: anti-CCL3-APC, anti-IFN-α-PE, and rat-anti-IL-10-Pe/Cy7 (Biolegend). For detection of the intranuclear marker NR4A1, the following conjugated human anti-mouse monoclonal antibody was used: anti-Nurr77 (NR4A1)-PE (Miltenyi Biotec). This human REA clone anti-mouse Nurr77 (NR4A1) IgG1 antibody cross-reacts with human NR4A1 as specified by MACS Miltenyi Biotec. We have previously validated this reagent [18]. Intracellular labeling was performed using the Cytofix/Cytoperm Fixation/Permeabilization kit and perm/wash buffer (BD-Biosciences). Intracellular non-specific binding sites were blocked using perm/wash buffer containing 20% hi-FBS, 50% rat serum and 20 ug of mouse IgG. Intranuclear labeling was performed using the FoxP3/Transcription Factor Staining Buffer Set (Invitrogen by Thermo Fisher Scientific, CA, USA), and non-specific binding sites were blocked using 20% hi-FBS. Cells were kept at 4 °C in 1.25% paraformaldehyde for 18 h prior to analysis. Data acquisition of 5 × 10^4^ events per sample was performed with LSRFortessa (BD-Biosciences), and analysis was done with FlowJo 7.6.3 software (TreeStar, Ashland, OR, USA). All stainings were compared to that of fluorescence minus one (FMO) values and isotype controls. Anti-mouse Ig(κ) and anti-rat Ig(κ) Compbeads Plus (BD Biosciences) were used to optimize fluorescence compensation settings. CS&T beads (BD Biosciences) were routinely used to calibrate the LSRFortessa to exclude the possibility of instrument-related fluorescence intensity changes over time, and consistency was verified prior to each data acquisition session using application settings based on Rainbow beads (BD Biosciences).

**Cell Sorting of Total Blood Monocytes, RNA Isolation, and Sequencing.** PBMCs samples were thawed, washed with IMDM followed by 1× PBS, and processed for flow cytometry, as stated above. Cells were stained using the following conjugated mouse anti-human monoclonal antibodies: anti-CD19/CD3/CD56-BV650, anti HLA-DR-PE-Cy7, anti-CD14-BV786 (BD-Biosciences), and anti-CD11c-PE-Cy5.5 (eBiosciences). Total CD14+ monocytes were sorted using a FACSAriaIII apparatus (BD-Biosciences) and kept at −80 °C in trizol (Invitrogen Life technologies) prior to sequencing. Total monocytes were sent to IRIC’s Genomics Core Facility for RNA extraction, sequencing, transcriptomic profiling, and analysis. Libraries were prepared using Clontech Ultra Low RNA SMARTer v4 (Takara) and sequenced on a HiSeq2000. Genes with adjusted *p*-values based on false discovery rate (FDR) values <0.05 were considered to be differentially expressed. Gene expression levels were compared using raw read counts and the negative binomial distribution model implemented in DESEq2, a differential expression analysis package developed for R.

**Statistical analyses.** Data from HESNs were compared separately to those of HIV-uninfected 2.5–5-year CSWs, HIV-infected CSWs, and HIV-uninfected non-CSWs. The *p*-values used to infer statistical significance of difference between groups was determined by unpaired Student’s T-test when continuous variables were normally distributed or by the Mann–Whitney U test otherwise. The D’Agostino–Pearson normality test was used to determine whether the values were sampled from a Gaussian distribution. Analyses were performed using R version 3.6.3 for Windows [19]. Data manipulations were done using the R packages tidyverse [20] and reshape2 [21]. Figures were produced using the R packages ggplot2 [22], ggfortify [23,24], gridExtra [25], ggpubr [26], and cowplot [27].

## 3. Results

### 3.1. Socio-Demographic Characteristics of the Study Groups

The socio-demographic characteristics of female CSWs and non-CSWs are shown in Table 1. There were no statistical differences for age between HESNs, HIV-1-uninfected 2.5–5-year CSWs “early HESNs”, and HIV-infected CSWs. There was a highly significant difference between the duration of sex work between HESNs, HIV-1-uninfected 2.5–5-year CSWs “early HESNs”, and HIV-infected CSWs. All women were practicing vaginal douching. The average number of clients and condom use were not significantly different between the CSWs groups.

### 3.2. Transcriptomic Analyses by RNA-Seq of Total Sorted Blood Monocytes from HESNs Reveal Distinct Effector and Regulatory Capacities

**Figure 1:**
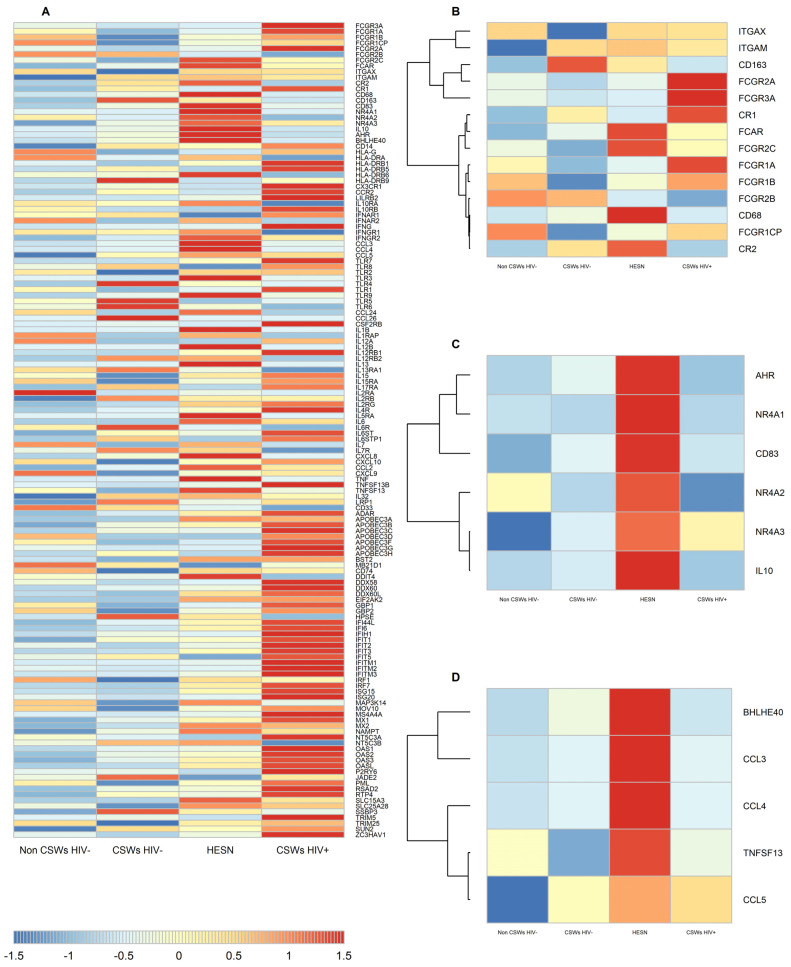
**Selected transcriptomic analyses by RNA-Seq of total sorted live blood monocytes.** (**A**) The heatmap shows total live monocytes expression levels of gene transcripts, the panel being selected based on our previous observations with the Beninese cohort [3,4,5] and the gene signature associated with protective vaccine regimen [28,29]. (**B**) Selected gene transcripts clustered for effector function. (**C**) Selected gene transcripts clustered for regulation markers. (**D**) Selected gene transcripts clustered for protection. Data are presented as the mean value of samples from three non-CSWs HIV- (HIV-1-uninfected control women from the general population), three CSWs HIV- (HIV-1-uninfected 2.5–5-year CSWs “early HESNs”), four HESNs (HIV-1-uninfected ≥ 10 years CSWs), and three CSWs HIV+ (HIV-1-infected CSWs).

Live total CD14+ monocytes were sorted from the PBMC samples of HESNs and relevant controls. RNA was extracted, purified, and submitted to a total transcriptomic analysis by RNA-Seq. Based on our previous observations with HESNs [3,4,5], and the gene signature associated with protective vaccine regimen [28], we herein present a heatmap selected for transcripts of interferon stimulated genes (ISG), Toll-Like Receptor (TLR) genes, genes associated with effector functions and protection/control against SHIV/HIV, and genes associated with tolerogenicity and immune regulation. As shown in Figure 1A, there is great variability between groups for this selected gene expression profile and *p*-values for statistical t-test between each group pairs are shown in Table S1. HESNs as well as early HESNs and women from the general population do not present a significant IFN signature, which is prevalent in the HIV-infected CSWs group. Albeit, that of HESNs is notable when compared to early HESNs and women from the general population. Strikingly, HESNs present increased expression levels of gene transcripts for the antibody Fc Receptors (FcR) FCGR2C and FCAR, as well as for complement binding receptors, such as ITGAX, ITGAM, and CR2 when compared to the other groups (Figure 1B). Transcripts for the Scavenger Receptors CD68 and CD163 genes were also increased for HESNs when compared to the other groups. Interestingly, monocytes from HIV-infected CSWs presented increased expression levels of gene transcripts for distinct FcRs such as FCGR3A, FCGR1A, FCGR1B, FCGR2A, and complement receptor CR1 when compared to HESNs and the other groups. Importantly, HESNs present increased expression levels of genes shown to be associated with protection/control against SHIV/HIV such as CCL3, CCL4, CCL5, the transcription factor basic helix-loop-helix family member e40 (BHLHE40), and TNFSF13, which encodes the growth factor A proliferation inducing ligand (APRIL), as well as with immune regulation such as IL-10, the IL-10 regulatory transcription factor aryl hydrocarbon receptor (Ahr), CD83, and the orphan nuclear receptors NR4A1, NR4A2, and NR4A3 when compared to the other groups (Figure 1C,D). Altogether, these observations suggest that blood monocytes of HESNs are endowed with a unique profile suggestive of a distinct effector, protective, and regulatory capacities when compared to the other groups.

### 3.3. Multicolor Flow Cytometry Analyses Expose Important Effector and Regulatory Capacities in Blood Monocytes from HESNs

**Figure 2:**
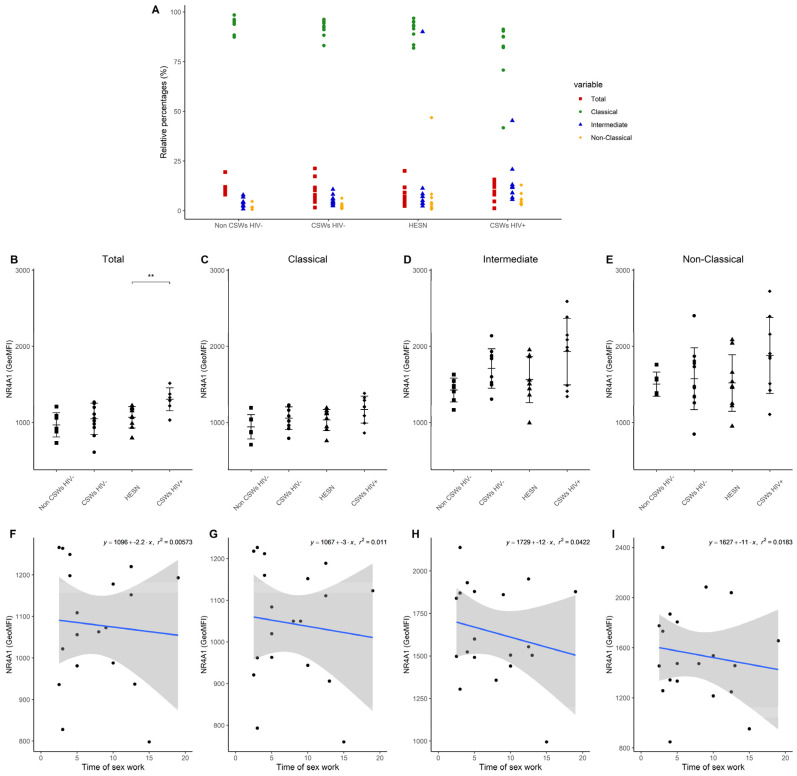
**Flow cytometry analyses of relative frequencies of live blood monocyte populations and NR4A1 protein expression levels.** (**A**) Relative frequencies of CD14+CD16− classical, CD14+CD16+ intermediate, and CD14^low/dim^CD16+ non-classical monocyte populations were calculated vs. total live CD14+ monocytes. (B–E) Levels of expression of NR4A1, as determined by geometric mean fluorescence intensity (GeoMFI), by (**B**) total, (**C**) classical, (**D**) intermediate, and (**E**) non-classical monocytes. Data are presented as the mean value ± SD of samples from 7 non-CSWs HIV- (HIV-1-uninfected control women from the general population), 9 CSWs HIV- (HIV-1-uninfected 2.5–5-year CSWs “early HESNs”), 11 HESNs (HIV-1-uninfected ≥ 10 years CSWs), and 9 CSWs HIV+ (HIV-1-infected CSWs). Significance levels are shown as * (*p* < 0.05), ** (*p* < 0.01), *** (*p* < 0.001). (**F**–**I**) Linear regression analyses are shown for CSWs HIV- (HIV-1-uninfected 2.5–5-year CSWs “early HESNs”) and HESNs (HIV-1-uninfected ≥ 10 years CSWs).

**Figure 3:**
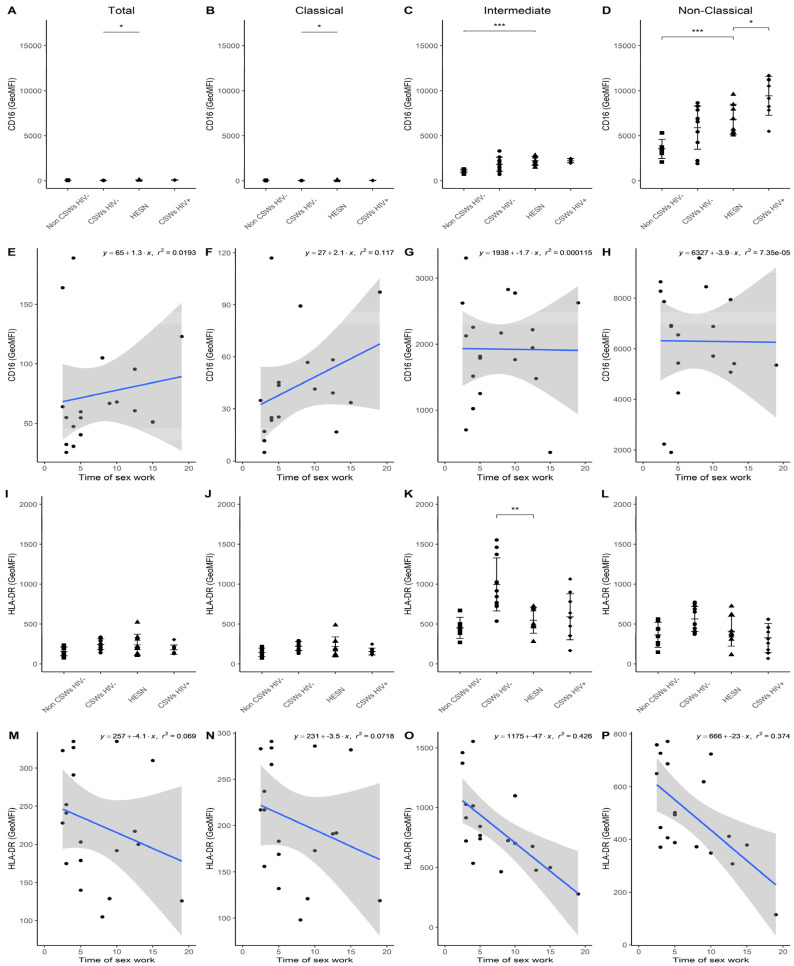
**Flow cytometry analyses of CD16 and HLA-DR expression levels by live blood monocyte populations.** (**A**–**D**) Levels of expression of CD16 and (**I**–**L**) HLA-DR proteins, as determined by geometric mean fluorescence intensity (GeoMFI), by (**A**,**I**) CD14+ total, (**B**,**J**) CD14+CD16− classical, (**C**,**K**) CD14+CD16+ intermediate, and (**D**,**L**) CD14^low/dim^CD16+ non-classical monocytes. Data are presented as the mean value ± SD of samples from 7 non-CSWs HIV- (HIV-1-uninfected control women from the general population), 9 CSWs HIV- (HIV-1-uninfected 2.5–5-year CSWs “early HESNs”), 11 HESNs (HIV-1-uninfected ≥ 10 years CSWs), and 9 CSWs HIV+ (HIV-1-infected CSWs). Significance levels are shown as * (*p* < 0.05), ** (*p* < 0.01), *** (*p* < 0.001). (**E**–**H**, **M**–**P**) Linear regression analyses are shown for CSWs HIV- (HIV-1-uninfected 2.5–5-year CSWs “early HESNs”) and HESNs (HIV-1-uninfected ≥ 10 years CSWs).

**Figure 4:**
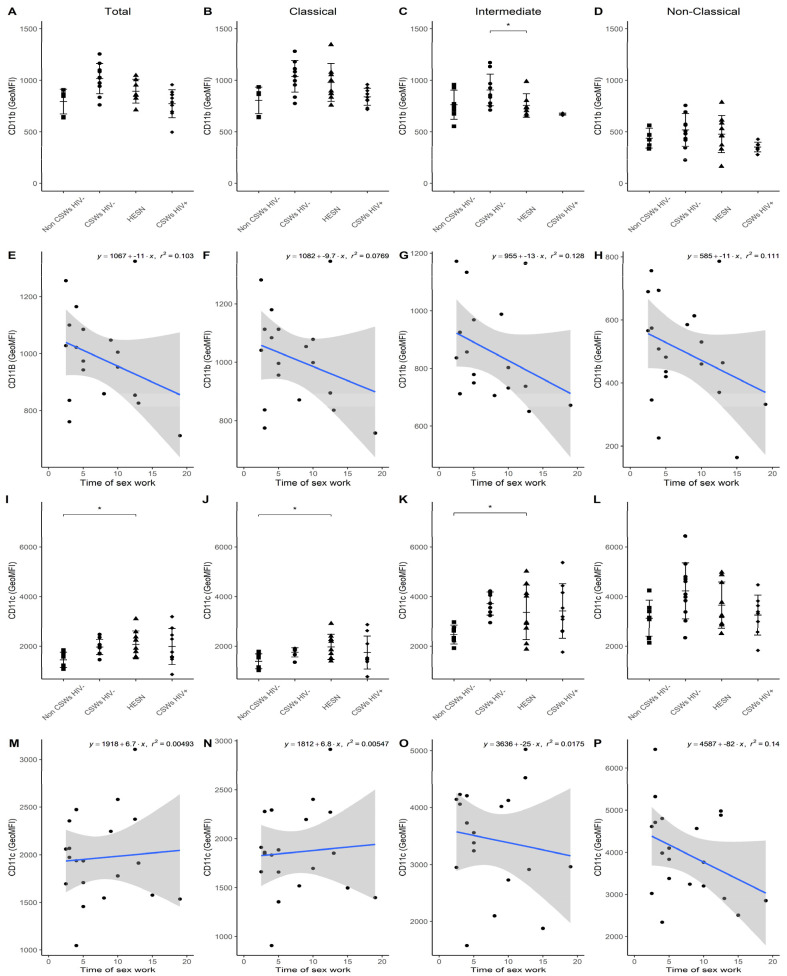
**Flow cytometry analyses of CD11b and CD11c expression levels by live blood monocyte populations.** (**A**–**D**) Levels of expression of CD11b and (**I**–**L**) CD11c proteins, as determined by geometric mean fluorescence intensity (GeoMFI), by (**A**,**I**) CD14+ total, (**B**,**J**) CD14+CD16− classical, (**C**,**K**) CD14+CD16+ intermediate, and (**D**,**L**) CD14^low/dim^CD16+ non-classical monocytes. Data are presented as the mean value ± SD of samples from 7 non-CSWs HIV- (HIV-1-uninfected control women from the general population), 9 CSWs HIV- (HIV-1-uninfected 2.5–5-year CSWs “early HESNs”), 11 HESNs (HIV-1-uninfected ≥ 10 years CSWs), and 9 CSWs HIV+ (HIV-1-infected CSWs). Significance levels are shown as * (*p* < 0.05). (**E**–**H**,**M**–**P**) Linear regression analyses are shown for CSWs HIV- (HIV-1-uninfected 2.5–5-year CSWs “early HESNs”) and HESNs (HIV-1-uninfected ≥ 10 years CSWs).

Similarly, to the idea described above, we performed multicolor flow cytometry analyses on blood monocytes of each group of participants by using selected panels of monoclonal antibody (mAb) cocktails targeting important markers we previously associated with effector, antiviral, and immunoregulatory properties in HESNs [3,4,5]. Total, as well as CD14+CD16- classical, CD14+CD16+ intermediate, and CD14low/dimCD16++ non-classical monocyte populations were assessed with these mAb panels. Gating strategies as well as the differential CCR2 and CX3CR1 expression profiles of monocyte populations are provided in Figures S1 and S2, respectively. Consistent with the elevated gene expression levels of NR4A1 (Figure 1), flow cytometry analyses demonstrate a trend towards slightly elevated relative frequencies of intermediate and non-classical monocyte populations, which highly express NR4A1 [14,15], in the blood of HESNs when compared to women from the general population (Figure 2A). This was also observed for HIV-uninfected 2.5–5-year CSWs “early HESNs” and HIV-infected CSWs. Moreover, NR4A1 expression levels tended to be slightly greater for these populations in HESNs, early HESNs, and HIV-infected CSWs when compared to women from the general population (Figure 2D,E), and the pattern of CD83 expression was similar (Figure S2). Upon analysis of CD16 (FCRG3A), we found that as for HIV-infected CSWs, intermediate and non-classical monocytes from HESNs and early HESNs presented elevated expression levels when compared to women from the general population (Figure 3C,D). Linear regression analyses show that this is more obvious in early HESNs when compared to HESNs (Figure 3G,H). In addition, HLA-DR surface expression levels tended to be more elevated in intermediate and non-classical monocytes of HESNs and early HESNS when compared to both HIV-infected CSWs and women from the general population (Figure 3K,L). As for CD16, linear regression analyses show that this is more obvious in early HESNs when compared to HESNs (Figure 3O,P). In a similar scheme, CD11b (ITGAM) protein expression levels were slightly elevated in all monocyte populations of HESNs and early HESNs when compared to both HIV-infected CSWs and women from the general population (Figure 4A–D). As for CD16 and HLA-DR, linear regression analyses show that this is more obvious in early HESNs when compared to HESNs (Figure 4E–H). CD11c (ITGAX) protein expression levels by monocyte populations of HESNs and early HESNs presented a similar profile to that of CD11b when compared to women from the general population and HIV-infected CSWs (Figure 4I–L). Linear regression analyses show that for intermediate and non-classical monocytes, this is more accentuated in early HESNs when compared to HESNs (Figure 4O,P).

**Figure 5:**
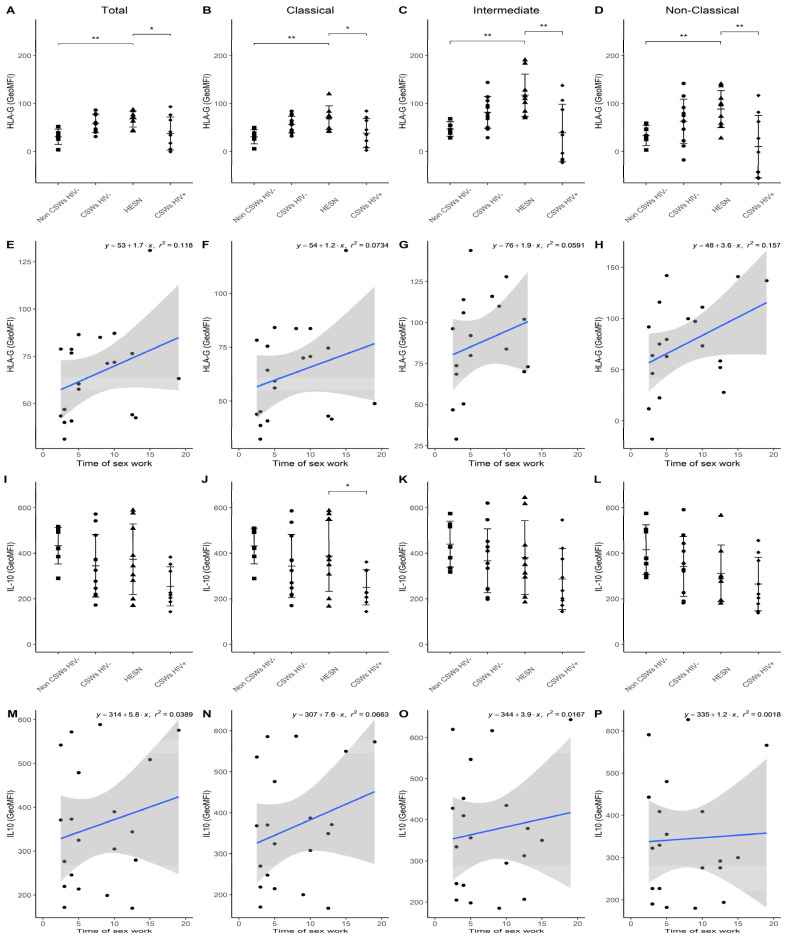
**Flow cytometry analyses of HLA-G and IL-10 expression levels by live blood monocyte populations.** (**A**–**D**) Levels of expression of surface HLA-G and (**I**–**L**) ex vivo IL-10 proteins, as determined by geometric mean fluorescence intensity (GeoMFI), by (**A**,**I**) CD14+ total, (**B**,**J**) CD14+CD16− classical, (**C**,**K**) CD14+CD16+ intermediate, and (**D**,**L**) CD14^low/dim^CD16+ non-classical monocytes. Data are presented as the mean value ± SD of samples from 7 non-CSWs HIV- (HIV-1-uninfected control women from the general population), 9 CSWs HIV- (HIV-1-uninfected 2.5-5 years CSWs “early HESNs”), 11 HESNs (HIV-1-uninfected ≥ 10 years CSWs), and 9 CSWs HIV+ (HIV-1-infected CSWs). Significance levels are shown as * (*p* < 0.05), ** (*p* < 0.01). (**E**–**H**, **M**–**P**) Linear regression analyses are shown for CSWs HIV- (HIV-1-uninfected 2.5–5-year CSWs “early HESNs”) and HESNs (HIV-1-uninfected ≥ 10 years CSWs).

Upon assessment of HLA-G surface expression levels, we clearly show that they are significantly increased by all monocyte populations of HESNs when compared to women from the general population and HIV-infected CSWs (Figure 5A–D). Although, we find a trend for this increase by monocytes from early HESNs, linear regression analyses depict that this is greater in the HESNs group (Figure 5E–H). Ex vivo IL-10 intracellular expression levels were comparable amongst all HIV-uninfected groups and tended to be higher than that observed on monocytes from HIV-infected CSWs (Figure 5I–L), especially when compared to classical monocytes from HESNs (Figure 5J). Linear regression analyses show that ex vivo IL-10 expression levels are greater for HESNs when compared to early HESNs (Figure 5M–P). Consistent with our data obtained through selected transcriptomic analyses, these flow cytometry results demonstrate that monocytes from HESNs bear important effector and regulatory capacities. We find that early HESNs are also endowed with a similar profile. These features seem to be shared characteristics of highly HIV-exposed individuals and are likely to confer an important advantage in their fight against HIV infection. Although, certain features observed in monocytes from HESNs and/or early HESNs were comparable to those of monocytes from HIV-infected CSWs, these are likely to reflect frequent HIV and/or microbial exposure.

### 3.4. Flow Cytometry Analyses Suggest That Monocytes and Plasmacytoid DC (pDC) Antiviral Capacities Are Preserved in HESNs When Compared to HIV-Infected CSWs

**Figure 6:**
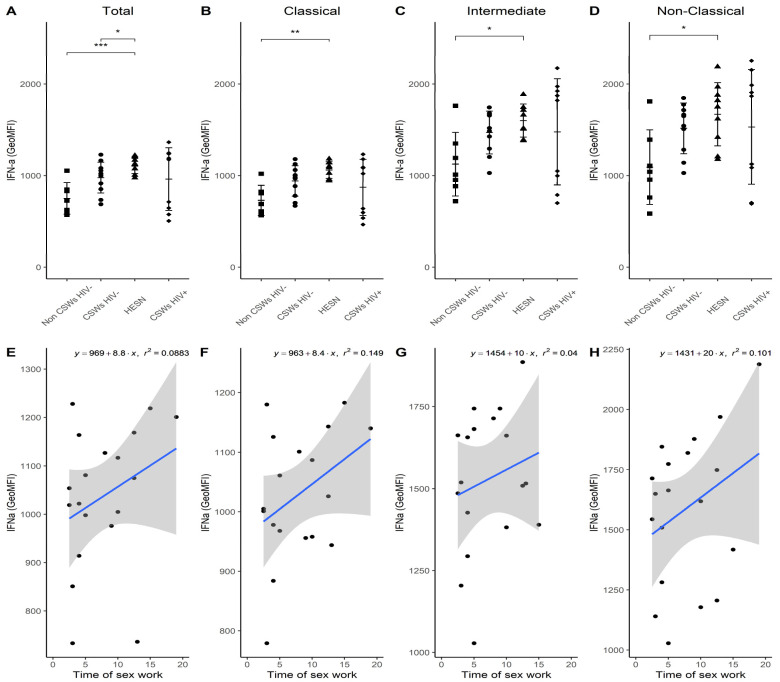
**Flow cytometry analyses of IFN-αexpression levels by live blood monocyte populations.** (**A**–**D**) Levels of expression of ex vivo IFN-α proteins, as determined by geometric mean fluorescence intensity (GeoMFI), by (**A**) CD14+ total, (**B**) CD14+CD16− classical, (**C**) CD14+CD16+ intermediate, and (**D**) CD14^low/dim^CD16+ non-classical monocytes. Data are presented as the mean value ± SD of samples from 7 non-CSWs HIV- (HIV-1-uninfected control women from the general population), 9 CSWs HIV- (HIV-1-uninfected 2.5–5-year CSWs “early HESNs”), 11 HESNs (HIV-1-uninfected ≥ 10 years CSWs), and 9 CSWs HIV+ (HIV-1-infected CSWs). Significance levels are shown as * (*p* < 0.05), ** (*p* < 0.01), *** (*p* < 0.001). (**E**–**H**) Linear regression analyses are shown for CSWs HIV- (HIV-1-uninfected 2.5–5-year CSWs “early HESNs”) and HESNs (HIV-1-uninfected ≥ 10 years CSWs).

**Figure 7:**
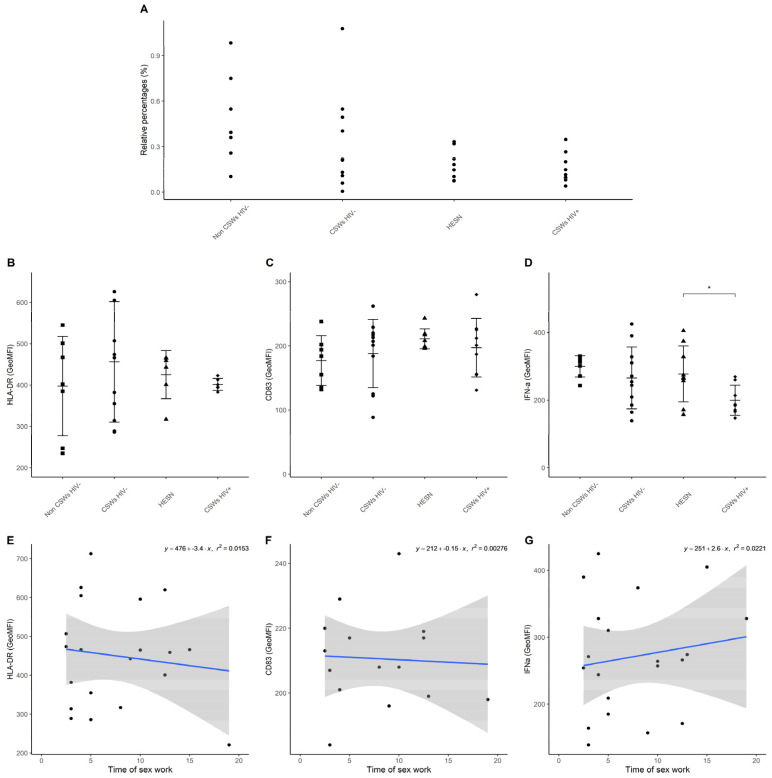
**Flow cytometry analyses of pDCs.** (**A**) pDC relative frequencies were calculated vs. total live PBMCs, (**B**) HLA-DR, (**C**) CD83, and (**D**) ex vivo intracellular IFN-α expression levels as determined by geometric mean fluorescence intensity (GeoMFI). Data are presented as the mean value ± SD of samples from 7 non-CSWs HIV- (HIV-1-uninfected control women from the general population), 9 CSWs HIV- (HIV-1-uninfected 2.5–5-year CSWs “early HESNs”), 11 HESNs (HIV-1-uninfected ≥ 10 years CSWs), and 9 CSWs HIV+ (HIV-1-infected CSWs). Significance levels are shown as * (*p* < 0.05). (**E**–**G**) Linear regression analyses are shown for CSWs HIV- (HIV-1-uninfected 2.5–5-year CSWs “early HESNs”) and HESNs (HIV-1-uninfected ≥ 10 years CSWs).

An assessment of ex vivo intracellular IFN-α expression levels demonstrates a significant increase by all monocyte populations from HESNs, and a trend for early HESNs when compared to monocytes from women of the general population (Figure 6A–D). Linear regression analyses show that this expression profile seems to drop with increasing years of sex work above 12 years (Figure 6E–H). We have taken advantage of our staining cocktails to track pDC relative frequencies (Figure 7A), and their HLA-DR expression levels (Figure 7B), CD83 expression levels (Figure 7C), and ex vivo intracellular IFN-α expression levels (Figure 7D) in the blood of HESNs and controls. We find that the latter is significantly elevated in HESNs when compared to HIV-infected CSWs (Figure 7D). This is in contrast to that observed for monocyte populations, whereby ex vivo intracellular IFN-α expression levels were not diminished for HIV-infected CSWs, the pattern clearly showing dichotomy amongst this group when compared to HESNs (Figure 6A–D). These results demonstrate that monocytes and pDC from HESNs, and to some extent early HESNs, are endowed with antiviral capacities, which are likely to contribute to their battle against HIV infection.

## 4. Discussion

We have characterized the blood monocyte compartment of Beninese HESNs and relevant controls (“early HESNs”, HIV-infected CSWs, low-risk HIV-negative women from the general population). Based on previous observations [3,4,5], we have herein concentrated our efforts on expression profiles of genes and proteins associated with effector functions, protection/control against HIV, antiviral capacities, and with tolerogenicity and immune regulation. Together, our transcriptomic and flow cytometry analyses provide strong evidence that total blood monocytes of HESNs are endowed with a unique profile suggestive of distinct effector, protective, and regulatory capacities when compared to the other groups.

Interestingly, a gene signature associated with partial protection in several non-human primates (NHP) vaccine trials [28] was recently reported in a human trial using the partially protective human RV144 vaccine regimen when compared to the non-protective human HVTN 505 vaccine regimen [30]. This enriched gene signature is associated with a decreased risk of HIV acquisition and increased vaccine efficacy. Importantly, transcriptomic analyses of PBMCs from vaccinees with the RV144 regimen [29] show that this gene signature is primarily expressed by myeloid cells, and especially monocytes, and involves molecules associated with effector functions, such as antibody-dependent cellular phagocytosis (ADCP), suggested to be a potential mechanism of vaccine protection. As such, robust ADCP responses were reported in the South-African RV144 vaccine trial [31]. In line with these observations, our data show that monocytes from HESNs present an increased expression of distinct FcRs as well as complement and scavenger receptors, all of which are solicited in the course of ADCP [32]. It is thus mandatory that we explore ADCP efficiency in our cohort. Of interest, HESNs present increased transcripts for the FCGR2C gene, encoding a low-affinity IgG FcR, which polymorphisms were shown to associate with HIV protection in the Thai RV144 vaccine trial [33]. Intriguingly, when a similar vaccine regimen was tested in South Africa in the HVTN702 trial, an allele shown to be protective in Thai vaccinees was rather significantly associated with increased odds of disease progression [34]. The Thai and South African populations are distinctly different at the FCGR2C gene locus [33,34], and whether polymorphisms confer protection in Beninese HESNs is of major interest and needs further attention.

Unlike monocytes from HESNs, we show that those from HIV-infected CSWs rather present increased gene transcripts for IgG-activating FcRs such as FCGR1A, FCGR2A, and FCGR3A, and this possibly reflects the overall inflammation we previously described for these individuals [2]. Consistently, upon assessment of CD16 surface expression levels (FCGR3A), we find non-classical monocytes from HESNs present lower levels when compared to that of HIV-infected CSWs, however still present significantly elevated when compared to women from the general population. This is compatible with our findings with blood NK cells of HESNs, which bear elevated surface levels of CD16 [35]. CD16 is required for antibody-dependent cell cytotoxicity (ADCC) by NK cells [36] and has been shown to be indispensable for ADCC activity by monocytes [37]. Interestingly, blood derived from IgG1- and IgG3-mediated ADCC activity toward the HIV Envelope (Env) V1V2 region was shown to correlate with protection in the Thai RV144 trial [38,39]. We could speculate that HESNs have strong ADCC capacities, which could contribute to their protection against HIV infection. However, when using highly sensitive and HIV-1 specific assays, we found no HIV-1 specific IgG mediating ADCC or neutralizing activities in the blood or genital samples of Beninese HESNs in contrast to HIV-infected CSWs [40]. It is possible, however, that ADCC activities in HESNs are mediated by IgG endowed with cross-reactivity, that were discarded for our assays. As such, we have detected some IgG1 with HIV Env gp41 subunit reactivity in genital samples of Beninese HESNs [41], which could be derived from cross-reactive, possibly first-line B-cell pools, as most gp41 Abs are known to cross-react with microbiota [42]. These observations may imply that natural immunity to HIV in Beninese HESNs is not mediated by HIV-1 highly-specific IgG neutralizing or ADCC responses, and may involve other Abs and/or responses, such as ADCP as stated above, that can confer some level of protection, as is now being suggested by a growing body of evidence [43,44,45].

As such, it was shown that in certain RV144 vaccinees, non-neutralizing IgA blocked in vitro binding of HIV Env glycoproteins to Galactosylceramide (GalCer), and mediated in vitro ADCP by monocytes [46]. In addition, it was recently shown that the gp41-specific broadly neutralizing antibody 2F5 under the IgA isotype (2F5-IgA), which triggers ADCC and cooperates with 2F5-IgG to increase HIV-1-infected cell lysis [47], induces ADCP not only of gp41-coated beads but also of primary HIV-1-infected cells in a FCRA1-dependent manner [48]. Although we report that monocytes from HESNs distinctively express increased gene transcripts for the IgA FcR FCRA when compared to the other groups, we did not assess HIV-1 reactivity of IgA in the blood of Beninese HESNs. Moreover, upon assessment of genital samples, we could not detect substantial IgA1 or IgA2 reactivity to HIV Env in HESNs [41]. To date, studies have reported contradictory results regarding anti-HIV-1 specific IgA responses in various cohorts with HESNs [2]. Discrepancies are likely to result from factors, such as the relatively small sample size and/or the different techniques used to detect Env-reactive Abs. In addition, the fact that most genital Igs are found in the mucus [49], may preclude substantial detection of certain Ig isotypes in genital samples. It is thus mandatory that we deploy further efforts in order to better characterize IgA reactivity in blood and genital samples from Beninese HESNs.

Overall, our data suggest that particular functional features of monocytes are likely to contribute to protection in Beninese HESNs. However, factors such as genetics, inflammatory status, opsonization length, frequency, and type of other phagocytes, as well as combination of expression of FcR and complement receptors, scavenger receptors, antibody specificity, isotype, subclass, and glycoforms may all influence the outcome of functional responses [32], and deserve further investigation.

Along with increased ADCP potential, the RV144-associated gene signature also showed that SEMA4A, SLC36A1, SERINC5, IL17RA, CTSD, CD68, and GAA were the most protective genes, and mostly expressed by monocytes [29]. This prompted us to investigate the levels of expression of these genes in our total monocyte RNA-Seq data bank. We found that along for the scavenger receptor CD68 (Figure 1), CTSD, and GAA (Figure S3) transcripts were significantly or tended to be increased, respectively, in monocytes from HESNs when compared to women from the general population. Moreover, gene transcripts for SERINC5 and GAA were significantly or tended to be increased, respectively, in monocytes from HESNs when compared to HIV-infected CSWs. Interestingly, TNFSF13, the gene encoding A proliferation-inducing ligand (APRIL), was found to be of the most protective genes in the NHP trials [28], and we found the expression levels of this gene to be increased in monocytes from HESNs when compared to the other groups. Of interest, APRIL has been associated with slower HIV disease progression in LTNP [50]. Therefore, our data suggest that natural immunity to HIV in HESNs shares some highly protective features elicited by vaccine regimens known to confer some level of protection in humans and NHP.

Our observations also suggest that the differential molecular profile of blood monocytes from HESNs reflects regulated functions, which is compatible with our previous observations in the FGT of these individuals [5]. As such gene transcripts encoding IL-10, as well as transcription factors involved in regulation of IL-10 such as BHLHE40 [51], recently shown to confer protection in the NHP trials [28], and Ahr [52] were increased in monocytes from HESNs when compared to the other groups. Importantly, gene expression levels of NR4A1 were most pronounced for HESNs, as were those of NR4A2 and NR4A3 when compared to the other groups. The elevated expression levels of NR4A1-3 may allow for more regulated functions, as has been found for Tregs [53], and non-classical monocyte populations [14,15]. Furthermore, we have recently described that marginal zone precursor (MZp) B-cells highly express NR4As and are endowed with a Breg function, which involves CD83 signaling [18]. CD83 being a regulatory molecule [54], with its expression directly modulated by NR4As [55]. Growing evidence support that expression levels of NR4As are affected in pathogenic contexts [56], and synthetic regulation of NR4As expression is currently used for treating patients with certain leukemia/lymphomas [57] and could be envisaged for immunomodulatory purposes. In this view, it has been shown that increasing NR4A1 expression levels lead to diminished MoDC and T-cell activation profiles [58].

The increased NR4A1 gene transcripts we found in total monocytes from HESNs is consistent with the elevated frequencies of intermediate and non-classical populations, and their elevated NR4A1 protein expression levels [14,15]. However, in contrast to NR4A1 gene expression, these latter features were also observed for early HESNs and HIV-infected CSWs when compared to women from the general population. NR4As are early-induced transcription factors in response to a multitude of activating stimuli, and observations on gene transcripts vs. protein expression may involve several contributing factors [59], of which the identification is out of the scope of the present study.

Frequencies of intermediate/non-classical monocytes are expanded in blood in the context of several infectious diseases including HIV infection [12], and possibly frequent HIV exposure as suggested by our data. Moreover, this is also reported in the context of malaria [60], which is endemic in Benin and may help at the interpretation of our findings. Intermediate monocytes appear to bear elevated antigen presentation and inflammatory potential [61], while non-classical monocytes are known to sense nucleic acids and viruses via TLR7 and TLR8, and are important sources of CCL3, CCL5, and IFN-α [62,63]. They also have the capacity to generate anti-inflammatory tolerogenic responses via HLA-G/ILT4 [63]. These are consistent with our findings with HESNs. Although our RNA-Seq analyses show that gene transcripts encoding total HLA-G and LILRB2 (ILT4) are more pronounced in monocytes from HIV-infected CSWs when compared to HESNs, analyses by flow cytometry show significantly elevated expression levels of total HLA-G by all monocyte populations from HESNs, when compared to HIV-infected CSWs. This is consistent with the high levels of released soluble HLA-G, which we found in the blood of HIV-infected CSWs from the same cohort [64].

We believe alterations in the ratios of monocyte populations may dramatically influence MoDC-mediated immunity. In human, there are contradictory results as to whether non-classical monocytes differentiate into macrophages [65] or MoDCs [66] in vitro. MoDCs derived from non-classical monocytes presented a differential transcriptomic signature and expressed CD103, RALDH2, and TCF4 typical of mucosal DCs thought to play a role in mucosal homoeostasis [66]. Furthermore, in a model of transendothelial migration [67], non-classical monocytes preferentially acquired DC features. Suggesting that the elevated frequencies of non-classical monocytes bearing a regulatory/tolerogenic profile may confer an advantage to HESNs, by generating MoDCs with similar features.

The events involved in increasing frequencies of intermediate and non-classical monocytes presenting effector/regulatory and antiviral profiles in the blood of HESNs have yet to be determined and are likely to be multifactorial. As a result of the cross-sectional design, the present study cannot address whether the blood monocyte profile of HESNs has a protective role against HIV infection. Although comparison between HESNs and early HESNs, of which the sex work period goes from 2.5 to 5 years, controls to some extent the effects of sex work itself on monocyte immunology, a sex work period of four years was previously considered enough to confer an HESN status. We prefer to design these individuals as potentially evolving towards an HESN status. The total monocyte gene expression profile of these early HESNs differed from that of HESNs, and the flow cytometry analyses showed variation in time for certain markers, however the overall regulatory/tolerogenic profile was attributable to HESNs. Longitudinal studies and further phenotypic and functional characterizations are required to confirm markers with a protective role. Based on our observations, the differential molecular profile of blood monocytes from HESNs reflects enhanced effector, antiviral and regulatory functions, and seems concomitant with natural immunity against HIV. Harnessing such populations could lead to novel preventive strategies.

## Figures and Tables

**Table 1:** **Distribution of demographic and sexual behavior characteristics in HIV-1 uninfected non-CSWs, HIV-1-uninfected 2.5–5-year CSWs “early HESNs”, and HESNs and HIV-1 infected CSWs.** * *p*-value for comparisons between HESNs and the two other CSW groups were calculated with the Mann–Whitney U test for age and duration of sex work; Unpaired T-test for the number of clients; and Fisher’s exact test for condom use and vaginal douching. CSW, commercial sex worker; HIV, human immunodeficiency virus; HESN, HIV Highly-Exposed Seronegative; N, number of participants; NA, non-applicable; NS, nonsignificant; SD, standard deviation.

	Non CSWs HIV-	CSWs HIV-	HESN	CSWs HIV+	
	N = 10	N = 11	N = 10	N = 12	* *p*-value
Age, mean (SD), years	34 (6.5)	34 (7.6)	44 (7.9)	45 (9)	NS
Duration of sex work (SD), years	N/A	4 (0.9)	12 (3.7)	9 (4.5)	0.001337
Number of clients past week, mean (SD)	N/A	15 (14.5)	12 (14.4)	18 (22.5)	NS
Condom always used with clients past week	N/A	7	8	9	NS
Vaginal douching	10	11	10	12	NS

## Data Availability

The data presented in this study are available in the article and supplementary material.

## Supplementary Materials

The following supporting information can be downloaded at: https://www.mdpi.com/article/10.3390/v14020361/s1, Figure S1: Flow-cytometry gating strategy, Figure S2: Flow-cytometry analyses of CX3CR1, CCR2 and CD83 expression levels by live blood monocyte populations, Figure S3: RNA-Seq analyses of gene transcripts highly associated with protection in vaccine regimens; Table S1: RNA-seq p-values for t-test statistical analysis between each group.
