# Supplementary material for: Monocyte Gene and Molecular Expression Profiles Suggest Distinct Effector and Regulatory Functions in Beninese HIV Highly Exposed Seronegative Female Commercial Sex Workers
Source: Viruses. 2022 Feb 10;14(2):361. doi: 10.3390/v14020361 (PMC8878004; doi:10.3390/v14020361)

viruses

MDPI

Supplementary Table S1. RNA-Seq p-values for t-test statistical analysis between each group. The table shows comparison between total live monocytes expression levels of gene transcripts between groups. The genes shown are the same that were used to generate the heatmap on Fig.1. The statistical t-test analysis compares the average of each group and expresses the degree of similarity between the two groups compared on a range between 0 and 1.

Gene	T-test p-values					
	HESN vs Non CSWs HIV-	HESN vs CSWs HIV-	HESN vs CSWs HIV+	CSWs HIV- vs Non CSWs HIV-	CSWs HIV+ vs Non CSWs HIV-	CSWs HIV+ vs CSWs HIV-
MS4A4A	2,560E-02	7,435E-01	3,028E-02	5,175E-02	3,450E-05	1,257E-02
IFI6	4,444E-02	1,584E-01	3,887E-02	4,537E-01	1,152E-04	5,088E-04
CCL5	1,094E-04	2,317E-01	5,324E-01	4,833E-03	8,988E-04	5,675E-01
NAMPT	5,580E-05	1,756E-02	2,556E-01	5,648E-02	2,579E-03	2,157E-01
OASL	1,295E-01	2,484E-01	9,779E-02	6,275E-01	2,746E-03	4,944E-03
IL2RG	3,060E-02	1,582E-02	3,922E-01	9,951E-01	3,413E-03	1,072E-03
IL2RB	4,985E-03	8,256E-01	8,260E-01	2,763E-03	8,652E-03	6,597E-01
IFITM3	7,426E-01	7,353E-01	1,074E-03	5,282E-01	9,411E-03	3,078E-04
IFI44L	1,576E-01	5,836E-01	2,145E-01	3,560E-01	1,163E-02	7,353E-02
IFIH1	5,107E-01	2,611E-01	3,555E-04	7,279E-01	1,110E-02	1,414E-02
CX3CR1	1,315E-01	2,733E-02	6,030E-06	6,411E-01	1,106E-02	2,033E-02
NR4A3	1,796E-03	1,956E-01	4,441E-01	3,615E-02	1,184E-02	5,964E-01
OAS3	5,843E-01	9,938E-01	2,739E-02	5,891E-01	1,173E-02	2,682E-02
CCL3	7,410E-05	8,599E-02	1,107E-01	1,488E-02	1,096E-02	9,029E-01
IFITM2	5,327E-01	7,848E-01	4,899E-04	3,851E-01	1,260E-02	1,695E-04
LRP1	1,397E-01	8,741E-02	2,919E-01	2,647E-03	1,553E-02	5,123E-01
ISG15	4,973E-01	1,970E-01	1,341E-01	6,346E-01	4,358E-02	5,301E-03
SSBP3	1,930E-01	8,386E-01	5,191E-01	1,371E-01	4,677E-01	3,952E-01
GBP1	1,228E-01	6,602E-01	7,392E-02	5,281E-02	9,559E-01	2,597E-02
GBP2	3,506E-02	1,566E-01	6,055E-02	7,386E-04	6,679E-01	9,899E-04
MOV10	2,788E-01	1,465E-01	1,739E-01	1,716E-02	8,938E-01	4,917E-03
FCGR1B	2,824E-01	1,930E-01	4,383E-01	2,514E-02	7,028E-01	3,787E-02
FCGR1CP	3,272E-01	1,119E-01	6,466E-01	1,637E-02	5,688E-01	4,085E-02
FCGR1A	5,525E-02	6,112E-01	2,248E-02	1,765E-02	9,002E-01	5,243E-03
IL6R	4,713E-01	4,047E-01	6,365E-01	9,800E-01	2,529E-01	1,915E-01
ADAR	3,946E-01	4,468E-01	1,637E-01	8,643E-01	3,597E-02	3,123E-02
FCGR2A	1,829E-01	7,200E-01	2,675E-02	3,117E-01	5,157E-01	6,320E-02
FCGR3A	2,897E-01	7,975E-01	1,129E-02	4,069E-01	2,273E-01	2,277E-02
FCGR2C	1,342E-01	3,248E-02	4,014E-01	6,784E-01	4,547E-01	1,938E-01
FCGR2B	3,887E-01	1,488E-01	7,705E-01	6,664E-01	5,476E-01	2,485E-01
IL10	4,120E-01	3,395E-01	1,904E-01	9,749E-01	7,193E-01	7,143E-01
CR1	8,927E-01	2,444E-01	3,702E-02	2,395E-01	4,546E-02	3,564E-01
TLR5	1,612E-01	6,787E-03	2,228E-01	3,063E-01	7,565E-01	1,361E-01
RSAD2	7,172E-01	7,325E-01	1,008E-01	5,041E-01	6,731E-02	1,937E-01
EIF2AK2	9,945E-02	3,883E-02	8,988E-01	8,417E-01	7,816E-02	2,826E-02
IL1B	6,166E-04	3,526E-04	3,268E-04	8,192E-01	8,331E-01	9,841E-01
NR4A2	7,138E-02	7,455E-02	8,764E-03	8,346E-01	5,879E-01	4,014E-01

BHLHE40	1,088E-04	1,233E-02	1,475E-03	1,024E-01	3,042E-01	4,981E-01
CCR2	1,833E-01	5,755E-02	4,021E-03	7,126E-01	2,138E-01	3,278E-01
IL12A	2,130E-01	6,534E-01	2,167E-02	3,885E-01	3,755E-01	5,346E-02
RTP4	7,810E-01	7,480E-01	3,055E-01	9,925E-01	5,225E-01	4,806E-01
IL1RAP	8,267E-01	2,354E-01	2,279E-01	3,990E-01	3,893E-01	9,839E-01
TLR1	2,863E-01	9,285E-01	1,182E-01	2,510E-01	7,398E-01	9,807E-02
TLR6	6,440E-01	2,909E-01	4,997E-01	6,291E-01	2,858E-01	8,310E-02
CXCL8	4,187E-03	2,065E-01	7,634E-02	8,283E-02	2,008E-01	6,106E-01
CXCL9	2,726E-01	3,496E-01	7,330E-01	5,302E-02	4,282E-01	2,017E-01
CXCL10	3,714E-02	2,427E-01	8,159E-02	1,739E-03	5,992E-01	3,616E-03
HPSE	8,247E-01	3,316E-01	8,894E-01	5,176E-01	9,226E-01	4,053E-01
IL15	4,562E-01	3,096E-02	3,842E-01	7,397E-03	9,733E-01	2,454E-03
TLR2	4,917E-01	2,201E-02	8,287E-01	1,734E-01	3,782E-01	1,217E-02
DDX60	4,250E-01	4,407E-01	1,265E-02	9,138E-01	1,516E-01	8,455E-02
DDX60L	2,969E-01	4,149E-02	4,311E-01	4,351E-01	8,059E-02	4,710E-03
IL7R	1,873E-01	9,027E-01	5,875E-01	2,263E-01	4,038E-01	6,742E-01
IL6ST	9,679E-01	4,854E-01	1,167E-01	5,061E-01	1,486E-01	3,826E-01
IRF1	9,843E-02	3,955E-02	7,975E-01	4,762E-04	1,547E-01	2,061E-02
JADE2	7,226E-01	9,023E-02	2,199E-01	2,456E-01	4,574E-01	6,401E-01
CD14	3,543E-01	5,716E-01	2,875E-01	1,521E-01	6,043E-02	6,185E-01
CD74	2,945E-01	5,439E-01	8,547E-01	1,116E-01	3,764E-01	4,295E-01
CD83	3,687E-03	5,532E-02	2,882E-02	2,339E-01	3,425E-01	7,874E-01
HLA-G	3,142E-01	7,188E-01	4,846E-01	1,835E-01	7,032E-01	2,890E-01
HLA-DRA	1,678E-01	8,044E-01	4,304E-01	1,094E-01	3,711E-02	5,887E-01
HLA-DRB9	9,829E-01	2,192E-01	5,466E-01	2,632E-01	5,749E-01	5,265E-01
HLA-DRB5	4,525E-01	3,909E-01	7,373E-02	1,289E-01	1,874E-02	3,522E-01
HLA-DRB6	8,988E-01	7,225E-01	6,292E-01	8,490E-01	7,606E-01	8,983E-01
HLA-DRB1	7,225E-01	9,617E-01	3,222E-01	6,906E-01	5,959E-01	2,992E-01
MB21D1	3,207E-01	5,561E-01	5,871E-01	6,404E-01	6,120E-01	9,640E-01
IFNGR1	4,080E-02	5,545E-01	1,556E-02	1,289E-01	9,060E-01	6,727E-02
AHR	5,085E-02	6,123E-03	5,659E-04	6,181E-01	2,579E-01	4,791E-01
IL6	1,072E-01	3,916E-02	8,138E-01	8,160E-01	1,591E-01	6,634E-02
NT5C3A	4,421E-01	3,659E-01	3,611E-03	9,678E-01	6,587E-02	4,395E-02
ZC3HAV1	4,044E-01	9,196E-01	2,018E-01	3,551E-01	7,578E-01	1,681E-01
DDX58	2,575E-01	6,128E-01	3,448E-02	4,964E-01	4,470E-01	1,072E-01
TLR4	5,166E-01	1,204E-01	8,234E-01	4,587E-01	6,534E-01	1,834E-01
IL15RA	6,190E-01	5,571E-01	1,948E-01	3,058E-01	5,070E-01	5,929E-02
IL2RA	2,103E-01	1,465E-01	6,179E-01	1,000E+00	4,108E-01	3,352E-01
DDIT4	5,156E-02	8,595E-02	5,780E-03	6,806E-01	6,014E-01	2,964E-01
IFIT2	3,232E-01	4,692E-01	6,517E-02	7,334E-01	5,080E-01	2,623E-01
IFIT3	8,418E-01	9,660E-01	4,268E-02	8,121E-01	1,067E-01	3,848E-02
IFIT1	7,185E-01	5,007E-01	1,193E-01	3,357E-01	7,955E-02	3,763E-01
IFIT5	1,136E-01	1,005E-01	2,155E-02	9,104E-01	6,332E-01	5,094E-01
SLC25A28	3,354E-01	3,769E-01	8,979E-01	7,927E-02	2,808E-01	4,499E-01
IFITM1	8,224E-01	8,320E-01	5,684E-03	6,787E-01	2,448E-02	2,899E-03
IRF7	7,218E-01	1,811E-01	1,957E-01	4,007E-01	1,302E-01	8,496E-03
TRIM5	8,130E-01	7,667E-01	5,792E-01	6,156E-01	4,637E-01	7,962E-01

SLC15A3	8,125E-02	1,031E-01	4,361E-01	7,744E-01	2,948E-01	3,946E-01
P2RY6	3,023E-01	5,075E-01	1,817E-02	6,605E-01	2,751E-01	8,701E-02
IL10RA	3,172E-01	2,919E-01	1,549E-02	9,541E-01	2,440E-01	1,716E-01
CD163	3,021E-01	2,578E-01	6,052E-01	4,095E-02	5,689E-01	9,923E-02
NR4A1	9,768E-02	4,249E-02	4,698E-02	8,743E-01	9,041E-01	9,664E-01
OAS1	5,462E-01	5,492E-01	4,162E-03	9,460E-01	4,999E-02	2,335E-02
OAS2	9,861E-01	6,384E-01	2,281E-02	6,616E-01	4,001E-02	7,076E-02
TNFSF13B	5,555E-01	8,598E-01	4,998E-02	6,659E-01	2,443E-01	7,427E-02
PML	4,330E-01	1,485E-01	1,719E-01	3,774E-02	6,613E-01	4,924E-03
ISG20	4,898E-01	8,222E-01	6,654E-02	3,722E-01	3,413E-01	3,930E-02
IL32	1,324E-01	7,802E-01	5,832E-01	2,077E-01	3,078E-01	7,869E-01
IL4R	9,995E-01	5,553E-01	8,982E-02	5,976E-01	1,285E-01	2,195E-02
ITGAM	2,902E-01	9,143E-01	8,584E-01	3,363E-01	3,691E-01	9,436E-01
ITGAX	8,441E-01	7,634E-02	7,217E-01	1,649E-01	9,029E-01	1,567E-01
TNFSF13	6,025E-01	1,889E-01	4,850E-01	5,123E-01	9,171E-01	5,381E-01
CD68	5,276E-02	1,341E-01	8,993E-02	5,418E-01	6,686E-01	8,391E-01
CCL2	1,988E-02	1,993E-01	7,053E-01	2,356E-01	4,632E-02	3,651E-01
NT5C3B	4,297E-01	7,466E-01	3,128E-01	6,159E-01	9,100E-01	4,918E-01
MAP3K14	1,338E-01	4,809E-03	8,442E-02	3,064E-01	9,648E-01	2,742E-01
TRIM25	6,664E-01	5,134E-02	8,574E-01	2,962E-02	7,869E-01	3,326E-02
BST2	5,098E-01	7,741E-02	7,883E-01	3,572E-01	3,684E-01	4,184E-02
IL12RB1	4,361E-01	6,327E-01	1,212E-01	2,269E-01	5,427E-01	4,225E-02
CD33	7,328E-01	6,829E-01	8,016E-01	9,808E-01	5,712E-01	5,092E-01
LILRB2	3,984E-01	7,489E-01	1,874E-03	2,580E-01	5,275E-02	6,032E-04
FCAR	2,017E-02	4,627E-02	1,695E-01	5,878E-01	2,734E-01	5,360E-01
IFNAR2	8,255E-01	4,818E-01	5,078E-01	6,826E-01	7,098E-01	9,673E-01
IL10RB	2,006E-01	6,415E-01	9,402E-03	3,873E-01	2,958E-01	3,269E-02
IFNAR1	3,366E-01	7,821E-02	3,736E-02	5,384E-01	3,665E-01	7,472E-01
IFNGR2	9,632E-03	1,497E-01	5,253E-01	1,932E-01	4,326E-02	4,205E-01
MX2	1,846E-01	1,343E-01	9,108E-01	9,903E-01	2,199E-01	1,660E-01
MX1	4,017E-01	8,102E-01	2,447E-01	5,328E-01	6,024E-02	1,604E-01
IL17RA	8,035E-01	2,004E-01	1,144E-01	3,699E-01	2,445E-01	7,652E-01
CSF2RB	2,984E-01	6,978E-01	4,485E-03	4,883E-01	1,328E-01	1,407E-02
SUN2	5,295E-01	8,842E-01	6,463E-01	4,476E-01	2,985E-01	7,538E-01
APOBEC3A	1,428E-01	5,561E-02	5,745E-01	8,055E-01	4,909E-02	1,330E-02
APOBEC3B	3,134E-01	5,244E-01	3,699E-01	6,595E-01	7,045E-02	1,247E-01
APOBEC3C	4,333E-01	5,146E-01	1,402E-02	8,409E-01	1,570E-01	7,073E-02
APOBEC3F	1,319E-02	5,484E-01	4,195E-03	5,131E-02	9,273E-01	2,280E-02
APOBEC3G	8,173E-02	3,634E-01	7,534E-03	3,530E-01	5,141E-01	7,708E-02
TLR7	4,507E-01	8,716E-01	1,922E-02	5,417E-01	1,799E-01	2,911E-02
TLR8	8,337E-02	1,053E-03	3,455E-04	2,301E-01	1,412E-01	7,615E-01
IL13RA1	8,748E-01	5,510E-02	1,585E-01	1,190E-01	1,554E-01	8,651E-04
IL12RB2	4,903E-01	9,460E-01	4,434E-01	5,279E-01	1,000E+00	4,836E-01
CR2	1,000E+00	8,858E-01	1,000E+00	8,706E-01	1,000E+00	8,622E-01
IL5RA	6,563E-01	5,977E-01	6,240E-01	1,000E+00	1,000E+00	1,000E+00
TLR9	1,145E-01	3,965E-01	4,297E-01	3,931E-01	3,663E-01	9,538E-01
TLR3	8,512E-01	7,154E-01	7,396E-01	8,895E-01	9,123E-01	9,745E-01

IL13	1,000E+00	1,000E+00	1,000E+00	1,000E+00	1,000E+00	1,000E+00
IL12B	3,461E-01	2,759E-01	2,932E-01	1,000E+00	1,000E+00	1,000E+00
TNF	NA	NA	NA	NA	NA	NA
CCL26	1,000E+00	8,858E-01	1,000E+00	8,706E-01	1,000E+00	8,622E-01
CCL24	9,391E-01	4,208E-01	4,432E-01	5,172E-01	5,396E-01	1,000E+00
IL7	5,621E-01	7,311E-01	8,719E-01	3,732E-01	4,680E-01	8,550E-01
IFNG	7,338E-01	8,433E-01	5,418E-01	8,706E-01	3,747E-01	4,175E-01
IL6STP1	1,000E+00	8,858E-01	8,565E-01	8,706E-01	8,445E-01	9,703E-01
CCL4	NA	NA	NA	NA	NA	NA
APOBEC3D	5,497E-01	9,696E-01	6,171E-01	5,243E-01	8,812E-01	5,876E-01
APOBEC3H	1,000E+00	5,083E-01	3,230E-01	5,327E-01	3,608E-01	7,404E-01

Supplementary Figure S1. Flow Cytometry Gating Strategy. Doublets were initially discarded through SSCW-SSCH and FSCW-FSCH gating. Lineage negative live cells were then selected, and total CD14+ monocyte populations were identified by $CD14^+CD16^-$ classical, $CD14^+CD16^+$ intermediate and $CD14^{low/dim}CD16^{++}$ non-classical profiles, which are CX3CR1 low to high, respectively. Live $CD14^-HLA\text{-}DR^+$ pDCs could be identified by their $CD11c^-CD123^+$ profile.

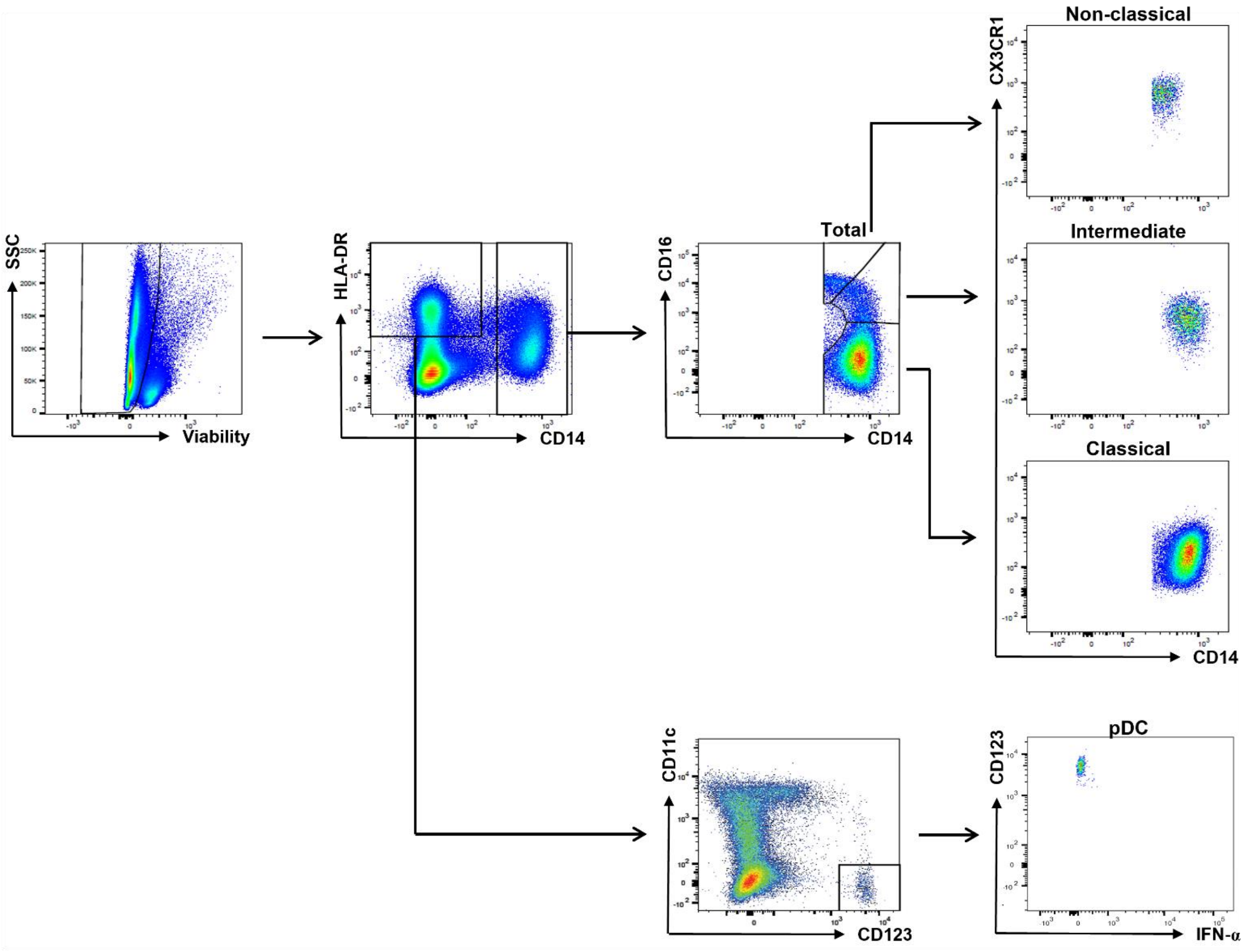

Supplementary Figure S2. Flow cytometry analyses of CX3CR1, CCR2 and CD83 expression levels by live blood monocyte populations. (A-D) Levels of expression of CX3CR1, **(E-H)** CCR2, **(I-L)** CD83 proteins, as determined by geometric mean fluorescence intensity (GeoMFI), by **(A, E, I)** $CD14^{+}$ total, (B, F, J) $CD14^{+}CD16^{-}$ classical, **(C, G, K)** $CD14^{+}CD16^{+}$ intermediate and **(D, H, L)** $CD14^{low/dim}CD16^{++}$ non-classical monocytes. Data are presented as the mean value ± SD of samples from 7 Non CSWs HIV- (HIV-1-uninfected control women from the general population), 9 CSWs HIV- (HIV-1-uninfected 2.5-5 years CSWs "early HESNs"), 11 HESNs (HIV-1-uninfected ≥ 10 years CSWs), and 9 CSWs HIV+ (HIV-1-infected CSWs). Significance levels are shown as * ($p < 0.05$), ** ($p < 0.01$), *** ($p < 0.001$).

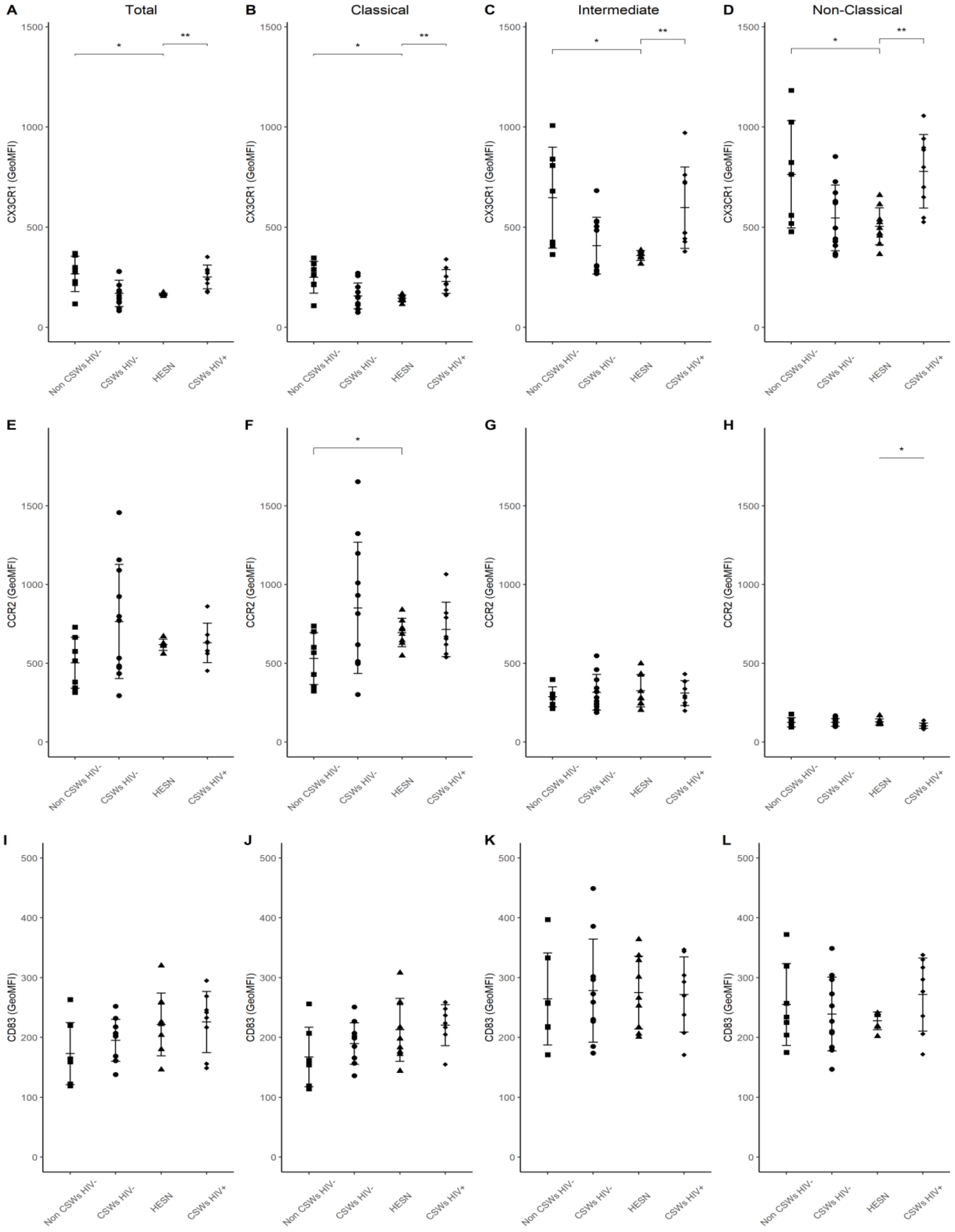

Supplementary Figure S3. RNA-Seq analyses of gene transcripts highly associated with protection in vaccine regimens. Gene expression levels of **(A)** SEMA4A, **(B)** SLC36A1, **(C)** SERINC5, **(D)** IL17RA, **(E)** CTSD and **(F)** GAA by total live CD14+ monocytes. Data are presented as the mean value of samples from 3 Non CSWs HIV- (HIV-1-uninfected control women from the general population), 3 CSWs HIV- (HIV-1-uninfected 2.5-5 years CSWs "early HESNs"), 4 HESNs (HIV-1-uninfected ≥ 10 years CSWs), and 3 CSWs HIV+ (HIV-1-infected CSWs). Significance levels are shown as * ($p < 0.05$), ** ($p < 0.01$), *** ($p < 0.001$).

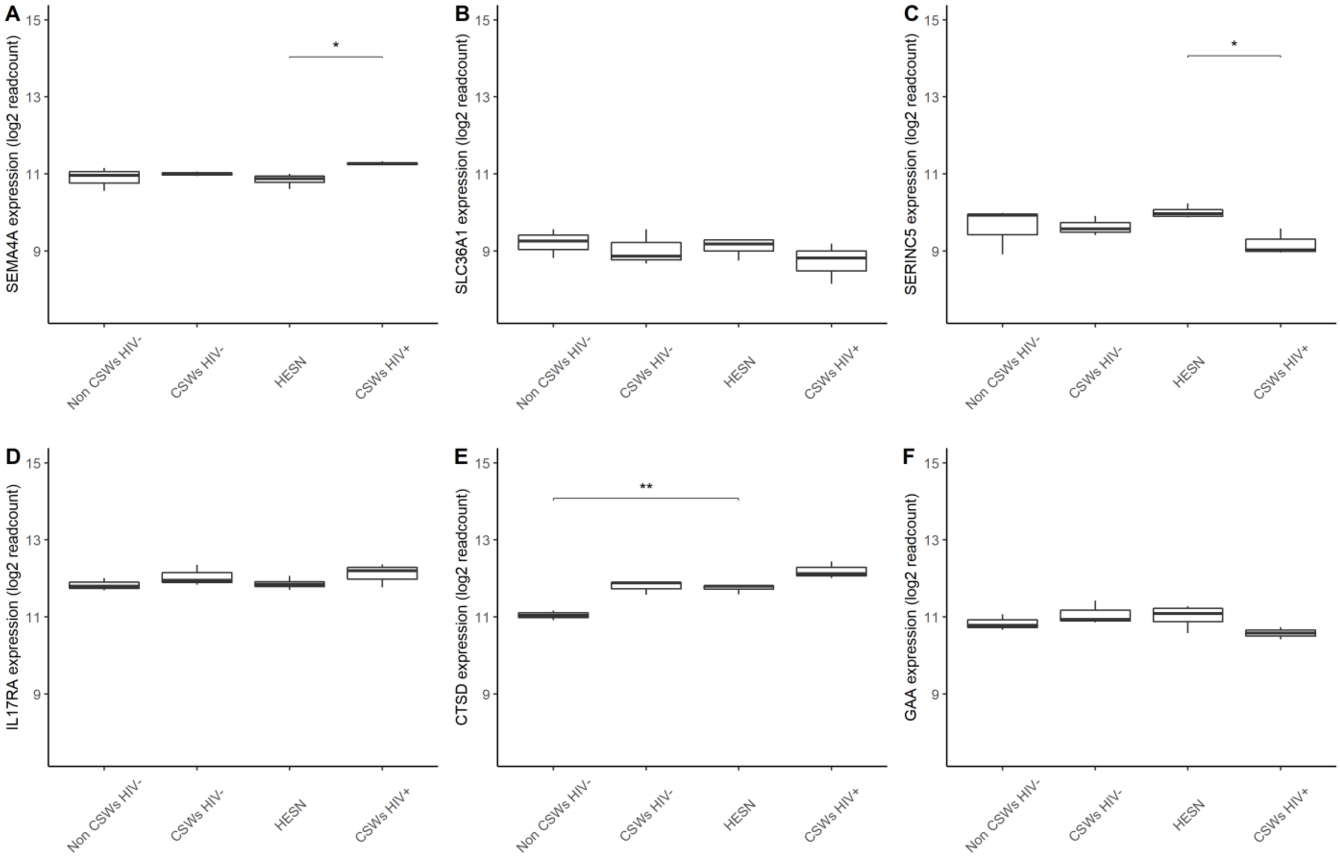

Supplement: Supplementary file 1 [file viruses-14-00361-s001.zip › viruses-1535809-supplementary.pdf]
